# Silver content dependent thermal conductivity and thermoelectric properties of electrodeposited antimony telluride thin films

**DOI:** 10.1038/s41598-019-45697-9

**Published:** 2019-06-25

**Authors:** Laia Ferrer-Argemi, Ziqi Yu, Jiwon Kim, Nosang V. Myung, Jae-Hong Lim, Jaeho Lee

**Affiliations:** 1 Department of Mechanical and Aerospace Engineering, University of California, Irvine, Irvine, CA 92697 USA; 2 Electrochemistry Research Group, Materials Processing Division, Korea Institute of Materials Science, Changwon-si, Gyeongnam 51508 Republic of Korea; 3 Department of Chemical and Environmental Engineering and UC-KIMS CIME, University of California-Riverside, Riverside, California 92521 USA; 4 Department of Materials Science and Engineering, Gachon University, Seongnam, 13120 Republic of Korea

**Keywords:** Thermoelectrics, Electronic devices

## Abstract

While electrodeposited antimony telluride thin films with silver contents demonstrated promising thermoelectric properties, their thermal conductivity and the silver content dependence remain unknown. Here, we report the thermal conductivities of Ag_3.9_Sb_33.6_Te_62.5_ and AgSbTe_2_ thin films with controlled annealing and temperature conditions and demonstrate the impact of silver content on thermal transport. After annealing at 160 °C, the room-temperature thermal conductivity of Ag_3.9_Sb_33.6_Te_62.5_ and AgSbTe_2_ thin films increases from 0.24 to 1.59 Wm^−1^ K^−1^ and from 0.17 to 0.56 Wm^−1^ K^−1^, respectively. Using phonon transport models and X-ray diffraction measurements, we attribute the thermal conductivity increases to the crystal growth and explain the thermal conductivity variations with the degree of crystallization. Unlike electrical properties reported in previous studies, the presence of silver contents has little impact on the thermal conductivity of Ag_3.9_Sb_33.6_Te_62.5_ and leads to a strong reduction in the thermal conductivity of AgSbTe_2_ thin films. By performing transient thermal conductivity measurements at 94 °C, we find the crystallization activation energy of Ag_3.9_Sb_33.6_Te_62.5_ and AgSbTe_2_ films as 1.14 eV and 1.16 eV, respectively. Their differences reveal the role of silver in inhibiting the nucleation and growth of Sb_2_Te_3_ crystals and impeding thermal transport. These findings provide guidance for optimizing doping and annealing conditions of antimony tellurides for near-room-temperature thermoelectric applications.

## Introduction

Nanostructured thermoelectric (TE) materials are widely studied for reducing the thermal conductivity and improving the power factor, which can increase the efficiency of thermoelectric power generators and solid-state cooling devices^1–5^. While fabrication of nanostructures often requires expensive and non-scalable processes, an electrodeposition technique provides combined attributes of cost-effectiveness, scalability, and precise control over crystallinity and composition^6–8^. Electrodeposition is thus ideal for studying the effects of crystal size and film composition, including doping concentration, in thermoelectric materials. Including dopants to the material composition has been proven to be an effective strategy to improve the thermoelectric figure of merit (*zT*) of chalcogenide semiconductors such as PbTe^9^, SnSb^10^, SnSe^5^, and AgSbTe_2_^11^. Doping can increase the electrical conductivity *σ* and have varying results on other properties. While low doping concentrations reduce the thermal conductivity *κ* and increase the Seebeck coefficient *S*, larger doping concentrations reduce the Seebeck coefficient and increase the thermal conductivity^1^. Yamashita *et al*.^12^ doped different antimony telluride compounds with tellurium excess to obtain *p*-type and *n*-type thermoelectric materials with a *zT* of 1.41 and 1.13, respectively. Mehta *et al*.^13^ measured an improved Sb_2_Te_3_ *zT* of 0.95 using sulfur doping and theoretically show that a maximum of 2 could be reached by doping optimization.

Among the antimony telluride material family, bulk AgSbTe_2_ has demonstrated an extremely low lattice thermal conductivity of 0.7 Wm^−1^ K^−1^ ^14^, which has been attributed to phonon scattering with heterophases^15^ and the formation of nanoscale cation sublattices^16^. Du *et al*.^11^ reached a *zT* of 1.4 at 550 K after doping AgSbTe_2_ with 2% Se. Concentrations of silver lower than the stoichiometry can produce impurities in the matrix or form precipitates, both of which are favorable to reduce the thermal conductivity^17,18^. In particular, Sb_2_Te_3_/Ag_2_Te composites fabricated using ball milling have been found to improve the *zT* up to 1.5 at 700 K, and AgSbTe_2_/Ag_2_Te quenched ingots reached 1.5 at 500 K^19^. Besides the material composition and doping, the morphology is been shown to be a key parameter in the resulting thermoelectric properties. For instance, our previous studies^6,8^ demonstrated a *zT* of 0.35 for electrodeposited Sb_37_Te_63_ films pre-annealed at 80 °C due to the combination of low thermal conductivity and enhanced power factor provided by a secondary phase; and nanostructured Bi_0.5_Sb_1.5_Te_3_ achieved a *zT* above 1 at room temperature by inducing small grain sizes and highly dense dislocations^20,21^.

Our recent studies reported the power factor of antimony telluride electrodeposited thin films with silver content from 0 to 30% and annealing temperatures up to 100 °C^7,22^. Among the studied electrodeposited thin films, the highest power factor (1870 μWm^−1^ K) was obtained in Ag_3.9_Sb_33.6_Te_62.5_ due to the Ag doping of the Sb_2_Te_3_ matrix and the presence of β-AgTe_2_ nanoprecipitates (~6% in vol.) that were formed after annealing at 100 °C^7^. Electrodeposited AgSbTe_2_ annealed at 100 °C also demonstrated a high power factor of 553 μWm^−1^ K and it is expected to present a very low thermal conductivity that could lead to higher *zT* values^14^. These power factors indicate promising materials for thermoelectric applications, which motivated a more detailed study of the thermal properties of the electrodeposited films with different compositions and annealing conditions. We report the thermal conductivity measurements of electrodeposited Ag_3.9_Sb_33.6_Te_62.5_ and AgSbTe_2_ thin films as a function of their pre-annealing temperature, annealing time, and measurement temperature. By comparing the thermal conductivity of SbTe films with varying silver contents and by analyzing the temperature- and time-dependent thermal conductivity data, this work provides a detailed understanding of the effects of silver content and crystallization on thermal transport.

## Results and Discussion

**Figure 1:**
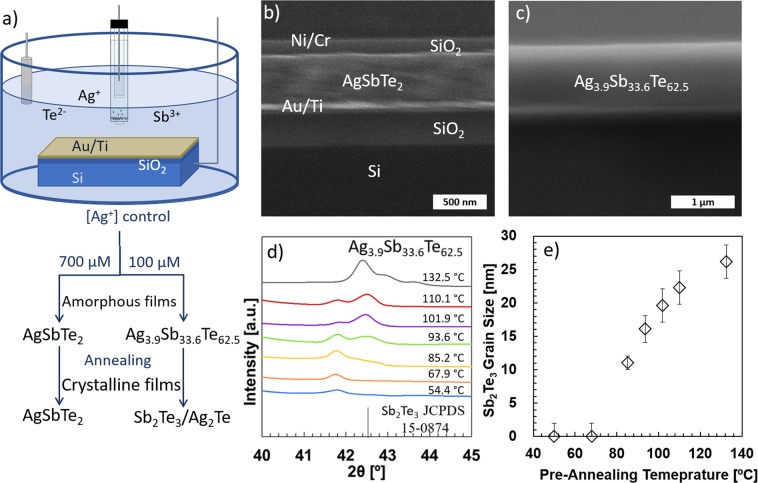
(**a**) Schematic of the sample fabrication by potentiostatic electrodeposition and control of the Ag^+^ concentration. The electrolytic exact composition can be found in our previous publications^7,22^. The amorphous electrodeposited films can be annealed at different temperatures to tune their thermoelectrical properties. A passivation layer (SiO_2_) and Ni/Cr electrodes are fabricated using e-beam deposition and photolithography^8^. Representative cross-sectional SEM images of measured AgSbTe_2_ (**b**) and Ag_3.9_Sb_33.6_Te_62.5_ (**e**) films. The thicknesses of the labeled layers of the Ni/Cr/SiO_2_/film/Au/Ti/SiO_2_/Si stack are 100 nm, 20 nm, 150 nm, 0.3–1 µm, 50 nm, 30 nm, 300 nm, and 500 µm, respectively. The AgSbTe_2_ film thickness ranged from 300 to 500 nm and the Ag_3.9_Sb_33.6_Te_62.5_ ranged from 600 to 1000 nm in our study. The fabrication processes are carefully controlled to keep the temperature below 50 °C and avoid any crystallization. (**c**) X-ray diffraction (XRD) data of an electrodeposited Ag_3.9_Sb_33.6_Te_62.5_ film after being annealed at 50, 68, 85, 94, 102, and 133 °C on a hot plate in vacuum for 30 minutes. Rhombohedral Sb_2_Te_3_ dominates the crystallization after annealing at 85 °C. AgSbTe_2_ films monotonically increase their crystal size with annealing temperature. (**d**) Average grain size of Sb_2_Te_3_ in Ag_3.9_Sb_33.6_Te_62.5_ films based on the XRD peak broadening, as estimated by the Debye-Scherrer equation.

The morphology and thickness of the electrodeposited AgSbTe_2_ and Ag_3.9_Sb_33.6_Te_62.5_ films are examined by Scanning Electron Microscopy (SEM) (Quanta, Thermo Scientific™) imaging. Figure 1(b) displays a cross-sectional SEM image of a measured AgSbTe_2_ film where we can identify, from top to bottom, the Ni/Cr electrode, the SiO_2_ passivation layer, the AgSbTe_2_ film, and the Au/Ti/SiO_2_/Si stack. The thickness of the AgSbTe_2_ films in our work ranges from 300 to 500 nm and the thickness of the Ag_3.9_Sb_33.6_Te_62.5_ films (Fig. 1(c)) ranges from 0.6 to 1 μm, all with compact and smooth morphology. The crystallinity and the phase transformation of AgSbTe_2_ and Ag_3.9_Sb_33.6_Te_62.5_ films are investigated by X-ray Diffraction (XRD) (Smartlab, Rigaku Corp™) analysis and the average grain size is estimated based on the major XRD peaks. Our previous studies confirmed the stoichiometry crystallization of AgSbTe_2_ in the Ag-rich film^22^, and the formation of β-Ag_2_Te (~6% in vol.) nanoprecipitates and a silver-doped Sb_2_Te_3_ matrix in the Ag-deficient film upon annealing at 100 °C^7^. In Fig. 1(d), XRD spectra of Ag_3.9_Sb_33.6_Te_62.5_ films pre-annealed at different temperatures shows a rapid Sb_2_Te_3_ crystallization (JCPDS 15-0874) after annealing at temperatures above 85 °C, reaching an average grain size of 26 nm after annealing at 133 °C. Detailed information on XRD spectra can be found in the Supplementary Note 1. The average grain size is estimated using the Debye-Scherrer equation and detailed in Fig. 1(e) as $\,D=\frac{k\lambda }{FWHM\cdot \,\cos \,\theta }$, where *k* is a shape factor whose value usually takes 0.9, *λ* is the X-ray wavelength of 1.54 Å, FWHM is the full-width-half-maximum obtained by analyzing XRD peaks, and *θ* is the Bragg angle. Peaks attributed to slightly deficient antimony telluride appears after annealing at 133 °C (Sb_0.405_Te_0.595_: JCPDS 45-1229) due to the precipitation of β-Ag_2_Te.

**Figure 2:**
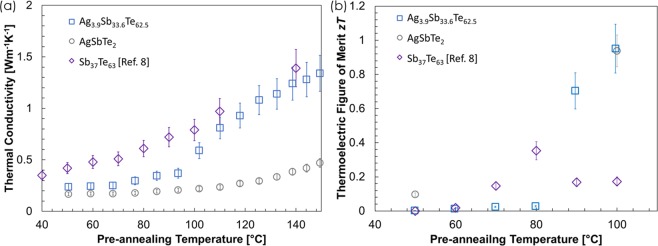
(**a**) Thermal conductivity of Ag_3.9_Sb_33.6_Te_62.5_ (blue square), and AgSbTe_2_ (grey circle) films measured using the 3ω method after annealing for 30 minutes in vacuum at different temperatures. The increasing trend is attributed to the increase in the films crystallinity and grain size as indicated by the XRD data. (**b**) Predicted thermoelectric figure of merit after annealing for 30 minutes. The power factor used in zT calculations is taken from our previous publications^7,22^. Our previous data on Sb_37_Te_63_ (purple diamonds) is also shown for comparison^8^.

Figure 2 shows the effective thermal conductivity *κ* as a function of the pre-annealing temperature, see the Methods section for details of the measurement technique. We observe a monotonic increase in the thermal conductivity of AgSbTe_2_ films which indicates increasing crystallization and larger phonon mean free paths; and, while Ag_3.9_Sb_33.6_Te_62.5_ films present only a slightly higher thermal conductivity at pre-annealing temperatures below 90 °C, a sudden increase occurs when Sb_2_Te_3_ starts to crystallize and reaches the same thermal conductivity as the Sb_37_Te_63_ reported in our previous study^8^. This indicates that the silver doping delays the crystallization of Sb_2_Te_3,_ providing a lower thermal conductivity at pre-annealing temperatures below 100 °C and that, unlike reported in previous studies of Sb_2_Te_3_ films^23^, the electron thermal conductivity does not contribute significantly to the total thermal conductivity of Ag_3.9_Sb_33.6_Te_62.5_ films which are 50 times more electrically conductive than Sb_37_Te_63_ films^6,7^. The overall lower thermal conductivity of the AgSbTe_2_ films is as expected because of the extremely low thermal conductivity of bulk AgSbTe_2_ (0.7 Wm^−1^ K^−1^) due to the strong anharmonicity of its bonding arrangement^24^. The main uncertainty of the thermal conductivity measured the 3ω method comes from the fitting of the temperature coefficient of resistance (TCR) to the measured resistance change with temperature, which is required for extracting the information about the thermal conductivity from the measured electrical data by matching the temperature rise with theoretical prediction given by the solution to the multilayer conduction equation. Although the resistance of the metal line is measured with a maximum uncertainty of 0.3% for the less resistive metal lines, and the placement of a platinum RTD sensor on the sample holder provides a more accurate temperature measurement with 2% uncertainty, the linear fit of the change of the resistance with temperature (dR/dT) leads to an uncertainty of up to 5%^25^. The TCR was first measured after annealing the sample at 70 °C and then another TCR was measured after annealing at higher temperatures. The TCR was consistent among samples (TCR_1_ = 0.005 ± 0.0002*K*^−1^) with the same annealing history, and no variations are expected before that because the resistance of the heater line at room temperature was constant. Another major uncertainty in our measurement is the thickness of the film, in which the contribution was minimized by performing cross-sectional SEM images. The film thicknesses were found to be constant within 10% in the measurement region as seen in Fig. 1. With the differential measurement, the only other uncertainty comes from the 3ω reading and fitting. The use of lock-in amplifier ensures accurate in-phase measurements within 0.5% and the fitting algorithm provides the best fit of the thermal conductivity down to 0.002 Wm^−1^ K^−1^. Overall, this results in an uncertainty between 15 and 20% for the highest and lowest thermal conductivity values, respectively. Using the power factors that we measured after different pre-annealing temperatures for samples of the same batch and reported in our previous work (i.e. 2.3 and 0.14 μWm^−1^ K^−2^ after annealing at 50 °C, and 1870 and 553 μWm^−1^ K^−2^ after annealing at 100 °C for Ag_3.9_Sb_33.6_Te_62.5_ and AgSbTe_2_ films, respectively)^7,22^, we obtain a maximum *zT* value at room temperature of 0.95 ± 0.17 for Ag_3.9_Sb_33.6_Te_62.5_ and 0.93 ± 0.10 for AgSbTe_2_ electrodeposited films after annealing at 100 °C. For details on the *zT* calculation, see Supplementary Note 2. The predicted *zT* values are among the highest reported to date among nanostructured bulk AgSbTe_2_^20,26–29^_,_ further experimentation is required to optimize the *zT* with slightly different pre-annealing temperatures around 100 °C.

**Figure 3:**
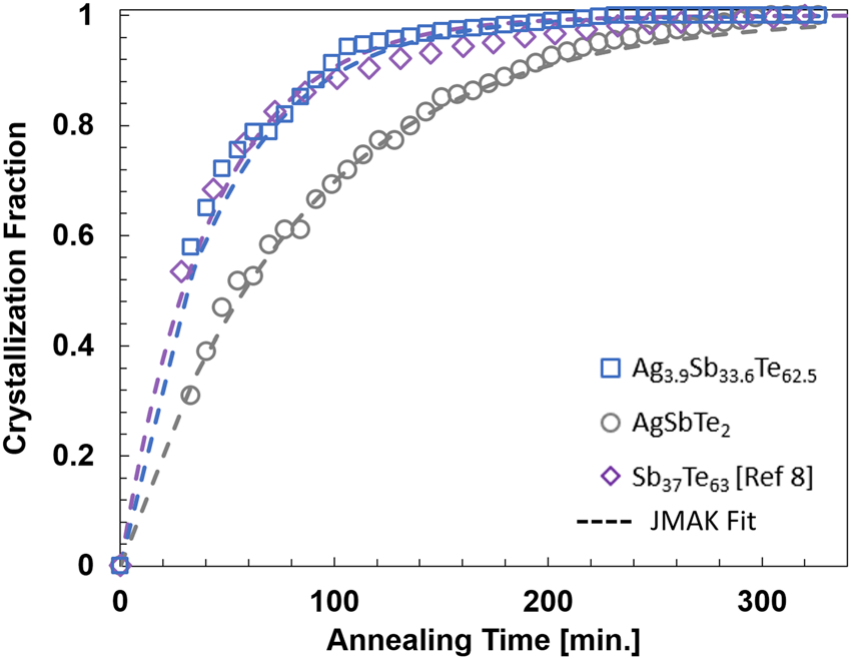
Crystallization fraction of Ag_3.9_Sb_33.6_Te_62.5_ (blue square) and AgSbTe_2_ (grey circle) films based on the thermal conductivity data as a function of annealing time at 94 °C. Data of Sb_37_Te_63_ (purple diamonds) from Yu *et al*. is also shown for comparison^8^. The data is fitted using the JMAK model (dashed lines) where the activation energy (1.14 eV for Ag_3.9_Sb_33.6_Te_62.5_ and 1.16 eV for AgSbTe_2_) and the Avrami constant (n = 1) are obtained by the best fit to the experimental data.

In order to compare the crystallization dynamics of AgSbTe_2_ and Ag_3.9_Sb_33.6_Te_62.5_ films, we measured the thermal conductivity during a 5.5-hour annealing at 94 °C. We convert the time-dependent thermal conductivity data into crystallization fraction by assuming that the amorphous regions are uniformly distributed across the film and in parallel with the crystallized regions, which is equivalent to assuming the columnar grain growth reported for other SbTe-based films^25^. Defining the stationary thermal conductivity reached at the end of the annealing process as crystalline thermal conductivity *κ*_*c*_ at the measured temperature and the amorphous thermal conductivity *κ*_*a*_ as the initial one, we can compute the crystalline fraction *x*_*c*_ from

1 $${\kappa }_{eff}={x}_{c}{\kappa }_{c}+(1-{{\rm{x}}}_{{\rm{c}}}){\kappa }_{a},$$

where *κ*_*z*_ is the effective cross-sectional thermal conductivity measured at each time step. The use of Eq. (1) for the volume fraction calculation assumes that the crystallized regions are uniformly distributed across the film in parallel with the amorphous regions. This is a reasonable assumption for films that have a columnar growth of crystalline regions upon thermal annealing^25^. Other effective medium theory models^30–35^ could be used with different assumptions on the crystalline phase distribution, but the change in volume fraction calculation is expected to be small. For instance, the use of another EMT assuming crystallized regions are distributed in series with amorphous regions results in a difference in the volume fraction calculation less than 0.046 for the transient thermal conductivity measurements performed in this work. In Fig. 3, we compare the crystallization fraction calculated from the experimental data with that predicted by the Johnson-Mehl-Avrami-Kolmogorov (JMAK) equation. The JMAK model^36^ relates the crystallization fraction with the crystallization activation energy Δ*H* and the time as ${x}_{c}=\exp (\,-\,{k}_{p}{t}^{n})$, where ${k}_{p}={\exp }(-{k}_{0}\frac{{\rm{\Delta }}H}{{k}_{B}T})$, *n* is the Avrami number, *k*_0_ is the crystallization constant, and *k*_B_ is the Boltzmann constant. We find a crystallization constant of 10^15^, a crystallization activation energy of 1.142 eV for Ag_3.9_Sb_33.6_Te_62.5_ films and 1.162 eV for AgSbTe_2_ films, and the Avrami number of 1, which indicates that the phase transformation process is governed by one-dimensional interface-controlled nucleation growth. The activation energy obtained for Ag_3.9_Sb_33.6_Te_62.5_ films coincides with the previously reported for Sb_37_Te_63_ films, which confirms the nucleation of Sb_2_Te_3_ and indicates that the creation of nanoprecipitates does not affect the crystallization rate. On the contrary, the activation energy required to crystallize AgSbTe_2_ is found to be slightly higher, which increases the annealing time required to achieve full crystallization and might be the reason why the crystallization of Sb_2_Te_3_ requires higher temperatures in the lightly silver doped films than in the previously studied Sb_37_Te_63_ films.

**Figure 4:**
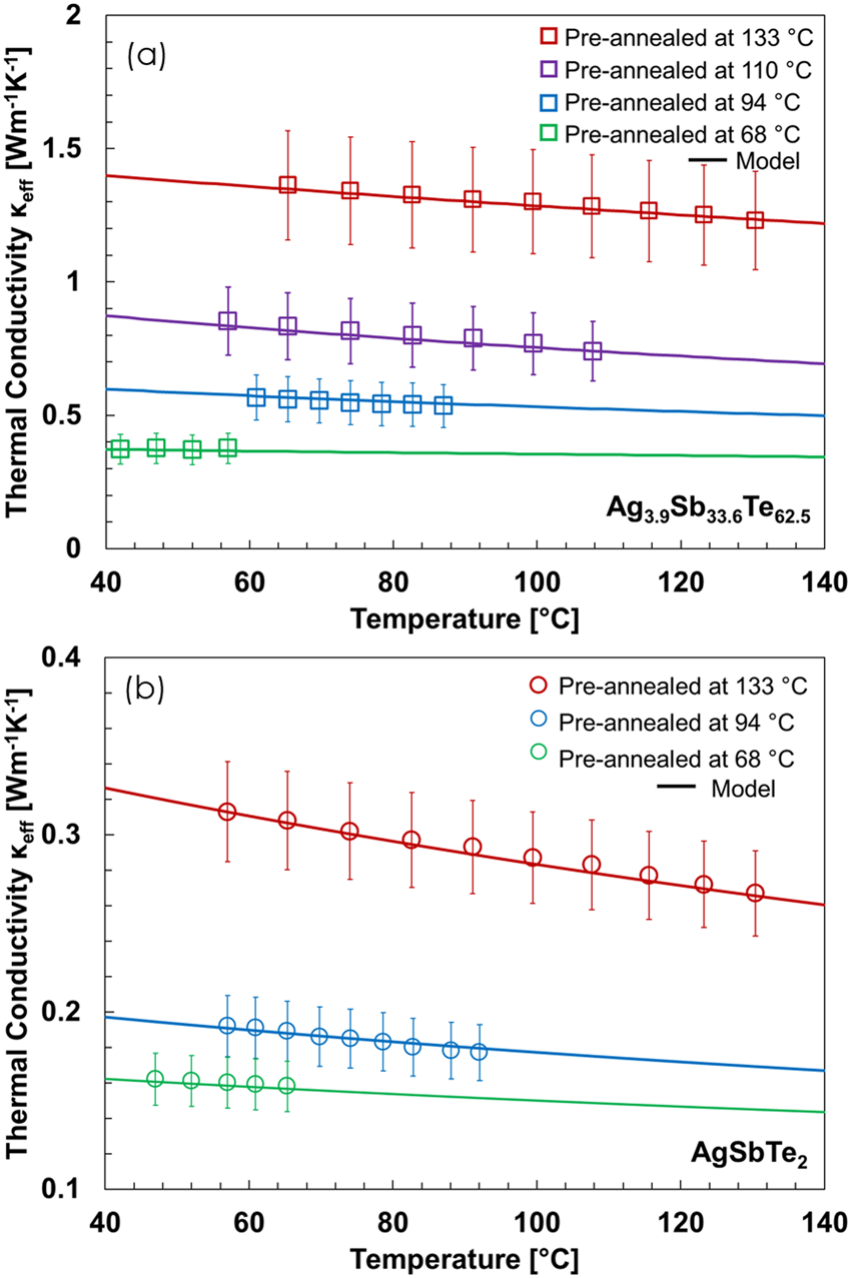
Thermal conductivity as a function of the measurement temperature for (**a**) Ag_3.9_Sb_33.6_Te_62.5_ and (**b**) AgSbTe_2_ films after being annealed at different pre-annealing temperatures. We use a combination of the Callaway model^44^ and the Einstein model^39^ to fit the thermal conductivity of the crystalline and amorphous phases, respectively. While Umklapp scattering behavior dominates after annealing at 133 °C due to the short phonon mean path of the Sb_2_Te_3_ and AgSbTe_2_ films, the weak temperature dependence of the films annealed at temperatures below 100 °C indicates a competing effect between amorphous and crystalline phases.

To confirm the crystalline nature of AgSbTe_2_ and Ag_3.9_Sb_33.6_Te_62.5_ films under different annealing conditions, we measured the temperature-dependent thermal conductivity of the films up to the pre-annealing temperature. The results, shown in Fig. 4, clearly show different temperature dependences after the films are annealed at different temperatures. The thermal conductivity of Ag_3.9_Sb_33.6_Te_62.5_ pre-annealed at 70 °C shows the typical behavior of a highly disordered amorphous material: an almost constant low value with a slight increase with temperature because of the thermal activation of thermal carriers with very short phonon average mean free path^37,38^. This trend can be supported by the modified Einstein model^39^, where a minimum thermal conductivity is described as the random walk of phonons through lattices and it is expressed as

2 $${\kappa }_{a}={(\frac{\pi }{6})}^{1/3}{k}_{B}{n}^{2/3}\sum _{i}{v}_{g}{(\frac{T}{{{\rm{\Theta }}}_{D}})}^{2}{\int }_{0}^{{{\rm{\Theta }}}_{i}/T}\frac{{x}^{3}{e}^{x}}{{({e}^{x}-1)}^{2}}dx,$$

where *n* is the atomic number density, the sum is taken over one longitudinal and two-transverse modes, *v*_*g*_ is the average sound velocity, *T* is the temperature of the film, and Θ_*D*_ is the Debye temperature for each mode. For AgSbTe_2_, the average sound speed, atomic number density, and Debye temperature are 1490 *ms*^−1^, 3.57 × 10^28^ *m*^−3^, and 150 K, respectively^40,41^. Due to the low volume fraction of Ag in Ag_3.9_Sb_33.6_Te_62.5_ films and the negligible difference between the atomic density and Debye temperature of AgSbTe_2_ and Sb_2_Te_3_ (3.11 × 10^28^ *m*^−3^ and 200 K, respectively) but the large disparity in average sound speed (2900 *ms*^−1^ for Sb_2_Te_3_)^42,43^, we use the latter as a fitting parameter for the data obtained after annealing Ag_3.9_Sb_33.6_Te_62.5_ films at 68 °C, obtaining an intermediate result of 2400 *ms*^−1^. The thermal conductivity of amorphous Ag_3.9_Sb_33.6_Te_62.5_ needs to be reduced by 20% to match the experimental results, a behavior that has already been observed in other complex amorphous materials^39^. As the films become more crystalline upon annealing, the enhanced acoustic properties improve the thermal conductivity and increase its dependency on the temperature. In crystalline structures, Umklapp scattering dominates the phonon transport at the temperature range of interest, which shortens the phonon mean free path and leads to a reduction in the thermal conductivity with the temperature. The thermal conductivity of crystalline samples *κ*_*C*_ is computed using the Callaway model^44^, which is expressed as

3 $${\kappa }_{C}=\frac{{k}_{B}}{2{\pi }^{2}}{(\frac{{k}_{B}T}{\hslash })}^{3}{\int }_{0}^{{{\rm{\Theta }}}_{D}/T}\tau \frac{{x}^{4}{e}^{x}}{{({e}^{x}-1)}^{2}}dx,$$

where the average phonon relaxation time *τ* is computed combining phonon-grain boundary ${\tau }_{GB}^{-1}=\frac{{v}_{g}}{3D/4}\frac{1-p}{p}$, film boundaries ${\tau }_{B}^{-1}=\frac{{v}_{g}}{t}$, phonon-impurity ${\tau }_{I}^{-1}=A{\omega }^{4}$, Umklapp ${\tau }_{U}^{-1}=B{\omega }^{2}{Texp}(-\frac{{{\rm{\Theta }}}_{D}}{T})$, phonon-carrier ${\tau }_{C}^{-1}=C\omega$, and, in the case of Ag_3.9_Sb_33.6_Te_62.5_ films, nanoprecipitate ${\tau }_{NP}^{-1}={v}_{g}{\rm{\Theta }}{n}_{NP}$ scattering mechanisms as ${\tau }^{-1}={\tau }_{GB}^{-1}+{\tau }_{B}^{-1}+{\tau }_{I}^{-1}+{\tau }_{U}^{-1}+{\tau }_{C}^{-1}+{\tau }_{NP}^{-1}$^45–47^, where *D* is the average grain size*, P* is the phonon transmission across the grain boundary, *t* is the thickness of the film, *n*_*NP*_ is the nanoprecipitate number density, and Θ is the average nanoprecipitate scattering cross-section, which is estimated using the average nanoinclusion size, and the mass and tensor strength difference with the matrix^17,48^. As detailed at the beginning of this section, the thickness of the film is measured using cross-sectional SEM imaging and the grain size is derived from the XRD spectra. The carrier scattering fitting parameter is taken as C = 8.2 × 10^−5^ ^49^ whereas the phonon-impurity and Umklapp scattering fitting parameters A and B are obtained from the best fit to our experimental data. We first find the impurity and Umklapp scattering parameters from Eq. (3) by fitting the data obtained after annealing at 133 °C and the amorphous phase from Eq. (2) by using the data obtained at the lowest annealing temperature; then, we find the crystalline fraction *x* that fits the data at the other temperatures. The fitting parameters result in ${{\rm{A}}}_{{{\rm{AgSbTe}}}_{2}}=9.0\cdot {10}^{-41}\,{{\rm{s}}}^{3}$, ${{\rm{A}}}_{{{\rm{Ag}}}_{3.9}{{\rm{Sb}}}_{33.6}{{\rm{Te}}}_{62.5}}=8.5\cdot {10}^{-42}\,{{\rm{s}}}^{3},{{\rm{B}}}_{{{\rm{AgSbTe}}}_{2}}=8.48\cdot {10}^{-15}\,{{\rm{s}}}^{-3},$ and ${{\rm{B}}}_{{{\rm{Ag}}}_{3.9}{{\rm{Sb}}}_{33.6}{{\rm{Te}}}_{62.5}}=1.32\cdot {10}^{-15}\,{{\rm{s}}}^{-3}$. These results show stronger dependency in impurity scattering than the previously reported Sb_37_Te_63_ films^8,49^. However, we noticed that the thermal conductivity of the Ag_3.9_Sb_33.6_Te_62.5_ films annealed below 133 °C cannot be fitted with such a strong impurity scattering component, and the best fitting results in ${{\rm{A}}}_{{{\rm{Ag}}}_{3.9}{{\rm{Sb}}}_{33.6}{{\rm{Te}}}_{62.5}}=3.3\cdot {10}^{-43}\,{{\rm{s}}}^{3}$ and ${B}_{{{\rm{Ag}}}_{3.9}{{\rm{Sb}}}_{33.6}{{\rm{Te}}}_{62.5}}=3.96\cdot {10}^{-15}\,{{\rm{s}}}^{-3}$. This indicates that the Ag_3.9_Sb_33.6_Te_62.5_ film might have started degradation when being annealed at 133 °C despite the SiO_2_ encapsulation. Alternatively, the weaker temperature-dependency after annealing at 133 °C might be due to the contribution of the electron thermal conductivity, which has a positive temperature dependence that can neutralize the negative temperature dependence of the lattice thermal conductivity^23^. Furthermore, temperature-dependent data after annealing at 161 °C was not possible due to degradation of the films at that temperature that caused leaking of the applied current. All Umklapp fitting parameters are lower than those previously obtained for Sb_2_Te_3_ films^8^ due to the stronger dependency on impurity scattering and the scattering by the nanoprecipitates. The coexistence of Sb_2_Te_3_ and β-Ag_2_Te nanoprecipitates in Ag_3.9_Sb_33.6_Te_62.5_ films does not produce a significant reduction in the thermal conductivity of the films due to the low volume fraction and similar thermal conductivity of both phases^50,51^. The results indicate that the crystalline fractions of the Ag_3.9_Sb_33.6_Te_62.5_ films annealed at 110, 94 and 68 °C are 0.45, 0.18 and 0, respectively; while those of AgSbTe_2_ films annealed at 94 and 68 °C are 0.1, 0.01, respectively. These crystalline fractions are lower than those previously obtained for Sb_37_Te_63_ films^8^, which further indicates that the presence of silver inhibits the nucleation of Sb_2_Te_3_ and AgSbTe_2_ crystals.

## Conclusions

We find the room-temperature thermal conductivity of electrodeposited AgSbTe_2_ thin films to be lower than that of the previously reported Sb_37_Te_63_ films^8^ due to the impeded thermal transport resulted from the inhibited nucleation and growth of Sb_2_Te_3_ crystals. The room-temperature thermal conductivity of electrodeposited Ag_3.9_Sb_33.6_Te_62.5_ films is lower that of Sb_37_Te_63_ films^8^ when the pre-annealing temperature is below 110 °C, and there is no significant difference when the pre-annealing temperature is above 110 °C. By annealing at 160 °C, the room-temperature thermal conductivities of Ag_3.9_Sb_33.6_Te_62.5_ and AgSbTe_2_ films increase from 0.24 Wm^−1^ K^−1^ to 1.59 Wm^−1^ K^−1^ and from 0.17 Wm^−1^ K^−1^ to and 0.56 Wm^−1^ K^−1^, respectively. We estimate the *zT* of 0.95 for Ag_3.9_Sb_33.6_Te_62.5_ and 0.93 for AgSbTe_2_ after annealing at 100 °C for 30 minutes by using the measured thermal conductivity values (0.59 Wm^−1^ K^−1^ for Ag_3.9_Sb_33.6_Te_62.5_ and 0.22 Wm^−1^ K^−1^ for AgSbTe_2_) from this paper and the measured power factors (1870 μWm^−1^ K^−2^ for Ag_3.9_Sb_33.6_Te_62.5_ and 553 μWm^−1^ K^−2^ for AgSbTe_2_) from the previous publications^7,22^, in which the samples were created from the same batch and treated with the same annealing conditions. The presented *zT* values at room are higher than any other Ag_*x*_Sb_*y*_Te_*z*_ films or nanostructures reported in the literature^11,26–29^. We attribute this to the unique phase control and the role of Ag in inhibiting nucleation.

The transient and temperature-dependent thermal conductivity measurements reveal that the silver content in Ag_3.9_Sb_33.6_Te_62.5_ films delays the formation of Sb_2_Te_3_ crystals but has little impact on the thermal conductivity, which is different from the large impact observed on electrical properties^7,22^. Our work shows that electrodeposited antimony telluride thin films with proper annealing conditions can be excellent thermoelectric materials, and AgSbTe_2_ is particularly promising due to the high crystallization activation energy and low thermal conductivity. These results improve our understanding of the role of metal content in chalcogenides for thermal transport and guide optimal designs of thermoelectric materials for near-room-temperature applications.

## Methods

Amorphous AgSbTe_2_ and Ag_3.9_Sb_33.6_Te_62.5_ films are prepared using the established electrodeposition method as schematized in Fig. 1(a) ^52–54^. The detailed process that allows well-controlled film composition by adjusting the applied potential and the electrolyte composition can be found in our previous publications^6,7,22^. After the amorphous Ag_*x*_Sb_*y*_Te_*z*_ thin films are deposited on SiO_2_ (300 nm)/Ti (30 nm)/Au (50 nm) films on a Si substrate, we deposit a 150 nm SiO_2_ layer by e-beam deposition to electrically passivate the films, and we fabricate the nickel electrodes required for the thermal conductivity measurements through photolithography patterning and electron beam evaporation of Cr (20 nm)/Ni (100 nm) films. The film temperature during all fabrication steps is limited to 50 °C to prevent any phase transition^6,7,22^.

The 3ω method^8,25,36,55,56^ is used to measure the cross-plane thermal conductivity. Briefly, in this method patterned metal lines, with a width ranging from 2 to 100 µm and a length ranging from 200 to 1000 µm, are used as both heaters and sensors. When an AC current (Keithley™ 6221 current source) at frequency ω causes Joule heating, it induces a temperature oscillation at 2ω, which results in a voltage drop across the heater at 3ω due to the metal characteristic electrical resistivity change with temperature^57^. The third harmonic of the voltage *V*_3ω_, which is recorded using a Stanford Research™ SR830 lock-in amplifier, is used to compute the temperature rise as ${\rm{\Delta }}T=\frac{2{V}_{3\omega }}{{V}_{1\omega }TCR}$, where the temperature coefficient of resistance $(TCR=\frac{1}{{R}_{0}}\frac{dT}{dR})$ is computed by the best fit to the measured resistance at different sample temperatures. The circuit used to measure the third harmonic is detailed in Fig. S2. The lock-in amplifiers are phase- and frequency-locked to the current source to avoid measuring the grid noise. To avoid saturating the lock-in amplifier with the first harmonic, an operational amplifier circuit is used to cancel the first harmonic, which is read by the multimeter and generated by the second lock-in amplifier in phase and at the same frequency. The temperature rise with a given applied current depends on how the underlying materials dissipate the heat generated and, therefore, is used to fit a multilayer heat conduction solution that accounts for the thermal resistance of each film, substrate, and relevant interfaces^56^. In order to accurately capture the thermal conductivity and thermal boundary resistances of the other layers (SiO_2_ and adhesion metal layers), we perform a differential measurement by measuring first the layer stack without the electrodeposited film. To identify the thermal conductivity variation with the film phase and crystallinity, the as-deposited amorphous AgSbTe_2_ and Ag_3.9_Sb_33.6_Te_62.5_ thin films are annealed at temperatures up to 160 °C for 30 minutes and then cooled down to room temperature to measure the thermal conductivity. The films then are heated on a hot plate using a Lake Shore™ 330 temperature controller in vacuum (Janis™ VPF-800) and a resistance temperature detector mounted on the chip holder provides an accurate temperature reading. Additionally, the formation kinetics of both materials is analyzed by annealing amorphous AgSbTe_2_ and Ag_3.9_Sb_33.6_Te_62.5_ films at 94 °C for 5.5 hours while measuring their thermal conductivity every 5 minutes with the 3ω method after the temperature of the sample stabilizes within 30 mK of the annealing temperature; and the heat transport mechanisms are studied using the temperature-dependent thermal conductivity after pre-annealing at different temperatures.

## Supplementary information

Supplementary Information

## Data Availability

The datasets generated during and/or analyzed during the current study are available from the corresponding author on reasonable request.
