# Supplementary material for: Silver content dependent thermal conductivity and thermoelectric properties of electrodeposited antimony telluride thin films
Source: Sci Rep. 2019 Jun 25;9:9242. doi: 10.1038/s41598-019-45697-9 (PMC6592942; doi:10.1038/s41598-019-45697-9)
Supplement: Supplementary file 1 — Supplementary Information [file 41598_2019_45697_MOESM1_ESM.pdf]

Supplementary Information

Silver content dependent thermal conductivity and thermoelectric properties of electrodeposited antimony telluride thin films

Laia Ferrer-Argemi,[a] Ziqi Yu,[a] Jiwon Kim,[b] Nosang V. Myung,[c] Jae-Hong Lim,*[b] and Jaeho Lee*[a]

[a]Department of Mechanical and Aerospace Engineering, University of California, Irvine, Irvine, CA 92697, USA.

E-mail: jaeholee@uci.edu

[b]Electrochemistry Research Group, Materials Processing Division, Korea Institute of Materials Science, Changwon-si, Gyeongnam 51508, Republic of Korea. E-mail: lim@kims.re.kr

[c]Department of Chemical and Environmental Engineering and UC-KIMS CIME, University of California-Riverside, Riverside, California 92521, USA

*Corresponding authors

Supplementary Note 1 – XRD Spectra

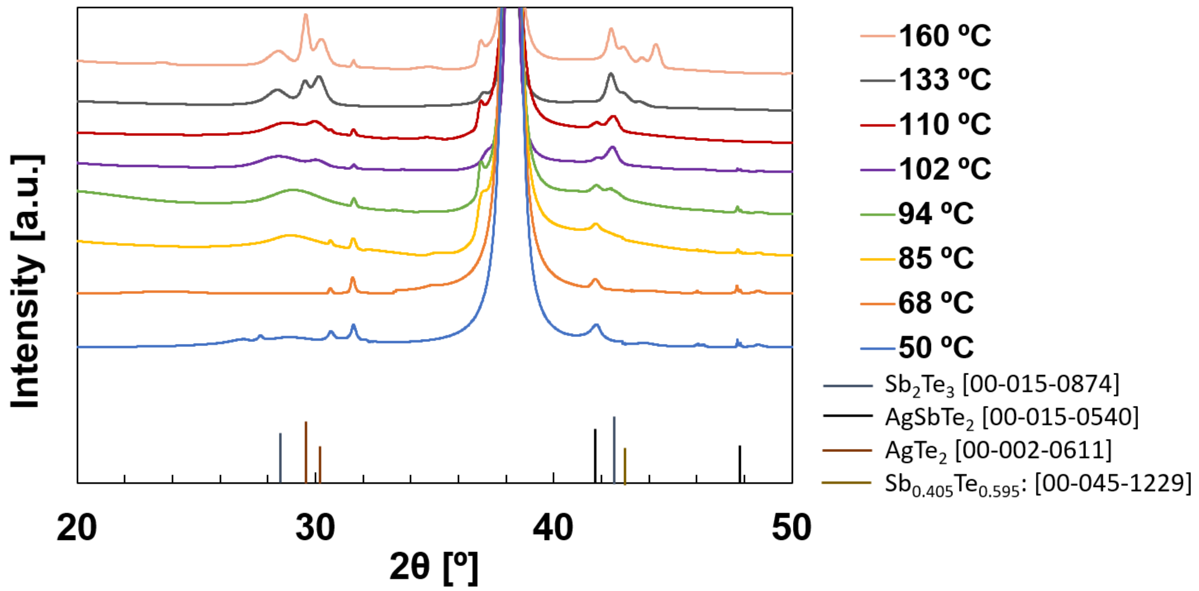

Fig. S1 XRD spectra of $Ag_{3.9}Sb_{33.6}Te_{62.5}$ films pre-annealed at different temperatures, the main peaks are identified as different compounds of silver, antimony and telluride. Sb_2Te_3 dominates after annealing temperatures of 90 ºC

Supplementary Note 2 – *zT* estimation

We can estimate the zT at room temperature using the electrical conductivity σ and the Seebeck coefficient S that we previously reported in $AgSbTe_2$ and $Ag_{3.9}Sb_{33.6}Te_{62.5}$ films fabricated

following the exact same procedure. The power factor of the $Ag_{3.9}Sb_{33.6}Te_{62.5}$ and $AgSbTe_2$ films after annealing at 100 ºC reached 1870 and 553 μ$Wm^{-1}K^{-2}$, respectively.[1,2] The annealing temperatures during the electrical measurements did not surpass 100 ºC due to the degradation of the films, but it was delayed during the thermal measurements by SiO_2 encapsulation. Using Wiedemann-Franz law to estimate the electron contribution to the thermal conductivity as $\kappa_e = 2.45 \cdot 10^{-8} \sigma T$,[3] where σ is the film electrical conductivity, we see that it is negligible up to after annealing at 90 ºC when the estimated κ_e =0.58 $Wm^{-1}K^{-1}$ becomes greater than the measured thermal conductivity value. This disparity may arise from the differences in annealing conditions: the electrical properties were reported after annealing in a tube furnace and we are reporting the thermal conditions after annealing on a hot plate in vacuum.[1,2] Moreover, the electrical data corresponds to in-plane measurements while we measure cross-plane thermal conductivity; hence, film anisotropy due to non-spherical grains can cause inaccuracies in the *zT* values. Given the previous columnar growth seen in GST films,[3] the in-plane thermal conductivity would be even lower, leading to larger *zT.* Taking the annealing differences into consideration and due to the large variation in the thermal conductivity of $Ag_{3.9}Sb_{33.6}Te_{62.5}$ films around the annealing temperature of interest, we conservatively estimate that the thermal conductivity of $Ag_{3.9}Sb_{33.6}Te_{62.5}$ and $AgSbTe_2$ films corresponding to the reported electrical measurements is 0.59 ± 0.16 $Wm^{-1}K^{-1}$ and 0.22 ± 0.02 $Wm^{-1}K^{-1}$, respectively. Even with the conservative estimations, the films still yield competitive maximum *zT* values at room temperature of 0.95 ± 0.15 and 0.93 ± 0.10 for $Ag_{3.9}Sb_{33.6}Te_{62.5}$ and $AgSbTe_2$ electrodeposited films, respectively.

Supplementary Note 3 – *3ω Measurement Circuit*

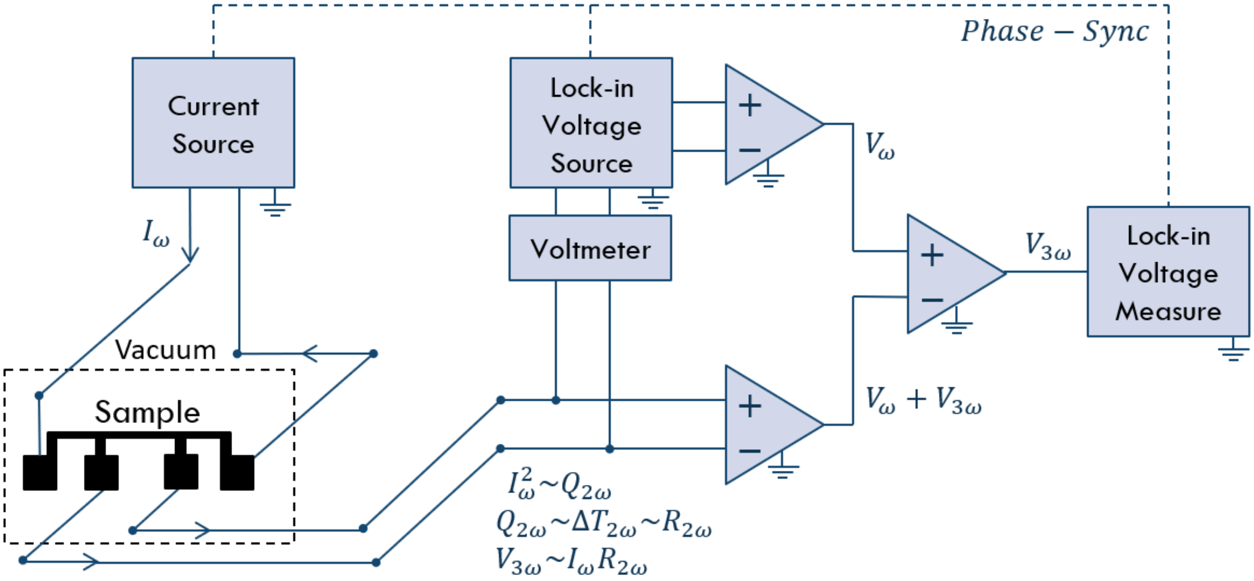

Fig. 5 Circuit used for the 3ω method measurement. All instruments are controlled with an in-house program through GPIB connections. The current is applied with a Keithley 6221 current source to the heater/sensor metal line fabricated on the sample, which is placed inside a Janis™ VPF-800 vacuum chamber. The generated first harmonic of the voltage drop is measured with a 34465A voltmeter and transferred to a SR830 lock-in amplifier, which cancels the first harmonic generated by the sample using an operational amplifier (AD8221) circuit. Another SR830 lock-in amplifier reads the third harmonic which is used to compute the thermal conductivity of the electrodeposited film. The current source and lock-in amplifiers are phase and frequency synchronized.

References

1. Kim, J., Lee, J.-Y., Lim, J.-H. & Myung, N. V. Optimization of Thermoelectric Properties of p-type AgSbTe2 Thin Films via Electrochemical Synthesis. *Electrochim. Acta* **196,** 579–586 (2016).

2. Kim, J., Lee, K. H., Kim, S.-D., Lim, J. & Myung, N. V. Simple and effective fabrication of Sb2Te3 films embedded with Ag2Te nanoprecipitates for enhanced thermoelectric performance. *J. Mater. Chem. A* **6,** 349–356 (2017).

3. Lee, J. *et al.* Thermal conductivity anisotropy and grain structure in Ge2Sb2Te5 films. *J. Appl. Phys.* **109,** 084902 (2011).
